# Antiretrovirals for Prevention: Realizing the Potential. Closing Commentary by the Executive Director of UNAIDS

**DOI:** 10.2174/157016211798038579

**Published:** 2011-09

**Authors:** Michel Sidibé

**Affiliations:** Joint United Nations Programme on HIV/AIDS (UNAIDS), Geneva, Switzerland

**Keywords:** Antiretrovirals, HIV, investment, HIV prevalence, HIV prevention, HIV treatment, HIV treatment for prevention, Treatment 2.0, UNAIDS.

## Abstract

Antiretroviral therapy (ART), for those who have access, has revolutionised the morbidity and mortality consequences of HIV infection. By the end of 2010, 6.6 million people living with HIV in low- and middle-income countries were receiving ART, a dramatic 20-fold increase since 2001, saving millions of lives. In addition to the impact of ART on the health of those living with HIV, recent randomised controlled trials demonstrate the additional impact of ART in reducing HIV transmission. With this double effect, ART is a game changer in the response to AIDS. With other advances over the past year, we now have a set of effective tools to stop the transmission of the virus and to keep people living with HIV healthy and productive. It is now the collective responsibility of researchers and implementers, of governments, the private sector and civil society, to identify and overcome the challenges and translate the science into real results for people. At the recent United Nations High Level Meeting on AIDS, Member States endorsed ambitious targets including to reach 15 million people living with HIV with ART and to cut sexual transmission of HIV by half by 2015. The declaration also calls for additional resources of 22 to 24 billion dollars by 2015 as an investment that will yield returns in multiples.

Over the past thirty years, we have seen Human Immunodeficiency Virus (HIV) infection transformed from a grim death sentence to a chronic manageable illness **—** at least in the medium term.

In 1987, zidovudine was introduced and created the first real hope that we could deal with this new pandemic. However, it wasn’t until 1996 that combination antiretroviral therapy (ART) truly changed the treatment landscape and produced the “Lazarus effect”**—**the ability to bring patients back from the brink of acquired immune deficiency syndrome (AIDS)-related death to seemingly full health [1].

However, the first drug combinations were extremely expensive, required frequent doses, often caused severe side effects, and resistance developed quickly due to imperfect adherence. These drawbacks, and a reluctance to provide the requisite finances, kept these medicines from broad international use.

Ten years ago, at the 2001 United Nations General Assembly Special Session on HIV/AIDS, consensus was not able to be achieved among world leaders to include treatment targets in the final Declaration of Commitment. At that time, only about 30,000 people in Africa had access to treatment [2].

## BREAKTHROUGHS BRING HOPE

Now, after years of intensive research and clinical experience, the quality, simplicity and tolerability of ART regimens have radically improved, and**—**thanks to the work of activists and competition from generic manufacturers **—** costs have fallen dramatically.

At the end of 2010, more than 6.6 million people living with HIV in low- and middle-income countries were receiving HIV treatment, and at the 2011 United Nations General Assembly High Level Meeting on AIDS, leaders agreed to the target of increasing this number to 15 million by 2015.

Today, we stand at the brink of a new way of thinking about the role of treatment, not just to save the lives of people who are infected, but to prevent infection in the first place **—** making the step from averting illness in individuals to drastically slowing the epidemic in populations.

Clear and unequivocal science has driven the agenda forward. Adding to prior observational and cohort studies, the *HIV Prevention Trials Network (HPTN) 052* randomised controlled trial demonstrated that early treatment for people living with HIV in serodiscordant couples can be 96% effective in preventing sexual transmission [3]. The *Partners Pre-exposure Prophylaxis (PrEP*) study findings demonstrated that pre-exposure prophylaxis, taken by an HIV-negative partner, can reduce sexual transmission by up to 73% [4].

These breakthroughs carry the promise of changing the context of HIV: tipping us from endemic HIV in perpetuity to an epidemic in decisive decline.

## REALITY CHECK

However, treatment for prevention is not a magic bullet. ART for prevention is an investment that needs careful nurturing so it can flourish as part of the portfolio of effective HIV prevention and care responses.

The challenge is to realise the potential of ART to help shift the dynamics of the global epidemic by adding its impact to other HIV prevention efforts and overcoming the global inequities that currently stand between impoverished people and their health, happiness and development.

The critical steps to tipping the HIV epidemic into decline revolve around simpler treatment, resolving the cost conundrum, placing communities at the centre of programme delivery and putting the whole prevention toolbox to use.

## SIMPLER AND SMARTER TREATMENT

With the drugs we currently have, there are strong and often serious side effects. Programmatic challenges continue, and in some countries, up to 40% of people on treatment are lost to follow-up within three years [5]. If HIV-symptomatic people have challenges to consistently taking their ART, with its complexity and side effects, it may be an even greater challenge to ensure that people who are without symptoms keep taking it.

The Joint United Nations Programme on HIV/AIDS (UNAIDS) and the World Health Organization (WHO) have developed a *Treatment 2.0* agenda: it calls for new pharmaceutical compounds that will lead to a “smarter, better pill” **—** less toxic, longer-acting, less expensive and easier to use. Combined with dose optimisation and improved sequencing of first- and second-line regimens, this will simplify treatment protocols, and**—**in concert with new models of service delivery, point of care testing and community mobilisation**—**will improve efficacy.

*Treatment 2.0* will not come to fruition unless we can reduce the time from research findings to policy implementation. The virus does not move slowly, and neither should we. To close the gap between science and implementation, we must be driven by a sense of urgency**—**the same sense of urgency felt by the millions who wait every day for discoveries to reach them.

## RESOLVING THE COST CONUNDRUM

The overall costs of providing HIV treatment are growing as countries scale up treatment, adopt the WHO guidelines for expanding early diagnosis and access to earlier treatment initiation, provide safer (but currently more expensive) first-line regimens and respond to the growing need for second- and third-line treatment.

Reducing the prices of antiretrovirals must continue to be given high priority. One effective route has been competition from generic drug producers, but this strategy has limited impact on the current prices for second-line treatment, as there are fewer second-line generic drugs available today. Another route to control costs is the Medicines Patent Pool, established in 2010, which aims to increase access to newer antiretrovirals by creating a pool of patents and intelligence on antiretroviral production donated by the originator companies.

The global consensus on trade regulation asserts the prior obligation of states to protect the public health, and accordingly as trade agreements are negotiated, we must challenge any components that seek to limit trade-related aspects of intellectual property rights (TRIPS) flexibilities. Countries must not succumb to pressure to amend intellectual property rights flexibilities or allow patent linkage, data exclusivity or patent term extension, or to form alliances that pre-empt countries’ use of compulsory licensing.

These measures to break the cost trajectory of treatment are among the answers to the skeptics who say that implementing treatment for prevention in this fiscal climate will be too costly, too risky and unsustainable. But what is truly costly, risky and unsustainable is inaction. If we want to turn scientific successes into progress for the poor, we must overcome the fiscal threats to HIV treatment and prevention access and the programmatic challenges to treatment adherence.

## COMMUNITY-CENTRED DELIVERY

Another critical pillar of sustainable treatment is ensuring that people and communities are at the centre of any approach. We need to go back to the community level to increase treatment literacy, increase demand for services and improve delivery on the ground. To make this possible, we must tap into unconventional capacity and introduce new alternative delivery systems. In that vein, UNAIDS is working closely with partners to mobilise more community health workers in Africa by 2015 [6].

Funders and policy makers must make sure that all people living with HIV and those most affected by the epidemic **—** particularly marginalised groups such as sex workers and their clients, men who have sex with men, people who inject drugs, and people in prison or other closed settings**—**can and will access integrated services that address their needs and protect their human rights. There is good evidence that effective outreach and engagement of affected communities will increase access, adherence and health outcomes.

## UTILISE THE ENTIRE PREVENTION TOOLBOX

We have already seen HIV prevalence among young people fall by more than 25% in 15 of the highest burden countries because of changes in sexual behaviour [7]. The strongest approaches are combination efforts, where biomedical, behavioural and structural changes reinforce one another. Among the new pieces of evidence for prevention effectiveness are the results from a South African study showing a 76% reduction in HIV incidence in circumcised men [8]. Maximising the potential of treatment for prevention requires it to be seen as a vital complement to other prevention efforts.

But there are still critical gaps in the “how” agenda, in terms of ensuring efforts are sensitive to national and local context, and that the most vulnerable and stigmatised groups do not miss out.

Currently, too much of the AIDS effort comes too late: many people are still only tested after they have been diagnosed with an opportunistic infection. While it may never be possible to eliminate acute stage infection as a driver of onward transmission, the gap between infection and HIV care can certainly be shortened, especially by eradicating the stigma and human rights violations that still deter health-seeking behaviour among key populations.

## KEEP OUR EYE ON THE PRIZE

Extending treatment access is at the centre of a new agenda of hope in the global AIDS response. Realising the full potential of antiretrovirals for prevention is a challenge for the scientific community, civil society and leaders everywhere.

But the prize is great. Modelling suggests that, compared with current treatment approaches, full implementation of smart HIV interventions, including *Treatment 2.0*, could avert an additional 10 million deaths by 2025 [9]. The question is not can we afford this now, but can we afford to pay both now, and forever, if we fail to make these smart and necessary investments?

Let us celebrate these scientific breakthroughs, and carefully nurture the investments that will maximise their full potential.

## ABBREVIATIONS

AIDS: = Acquired Immune Deficiency Syndrome
ART: = Antiretroviral Therapy
HIV: = Human Immunodeficiency Virus
HPTN: = HIV Prevention Trials Network
PrEP: = Pre-exposure Prophylaxis
TRIPS: = Trade-Related Aspects of Intellectual Property Rights
UNAIDS: = Joint United Nations Programme on HIV/AIDS
WHO: = World Health Organization
